# Mesenchymal Stem Cell-Derived Exosomes: Applications in Regenerative Medicine

**DOI:** 10.3390/cells10081959

**Published:** 2021-08-01

**Authors:** Mangesh D. Hade, Caitlin N. Suire, Zucai Suo

**Affiliations:** Department of Biomedical Sciences, College of Medicine, Florida State University, Tallahassee, FL 32306, USA; mangesh.hade@med.fsu.edu (M.D.H.); csuire@fsu.edu (C.N.S.)

**Keywords:** exosomes, extracellular vesicles, mesenchymal stem cells, regenerative medicine, microRNA, growth factors, wound healing, traumatic brain injury, cardiovascular disease, COVID-19

## Abstract

Exosomes are a type of extracellular vesicles, produced within multivesicular bodies, that are then released into the extracellular space through a merging of the multivesicular body with the plasma membrane. These vesicles are secreted by almost all cell types to aid in a vast array of cellular functions, including intercellular communication, cell differentiation and proliferation, angiogenesis, stress response, and immune signaling. This ability to contribute to several distinct processes is due to the complexity of exosomes, as they carry a multitude of signaling moieties, including proteins, lipids, cell surface receptors, enzymes, cytokines, transcription factors, and nucleic acids. The favorable biological properties of exosomes including biocompatibility, stability, low toxicity, and proficient exchange of molecular cargos make exosomes prime candidates for tissue engineering and regenerative medicine. Exploring the functions and molecular payloads of exosomes can facilitate tissue regeneration therapies and provide mechanistic insight into paracrine modulation of cellular activities. In this review, we summarize the current knowledge of exosome biogenesis, composition, and isolation methods. We also discuss emerging healing properties of exosomes and exosomal cargos, such as microRNAs, in brain injuries, cardiovascular disease, and COVID-19 amongst others. Overall, this review highlights the burgeoning roles and potential applications of exosomes in regenerative medicine.

## 1. Introduction

**Figure 1:**
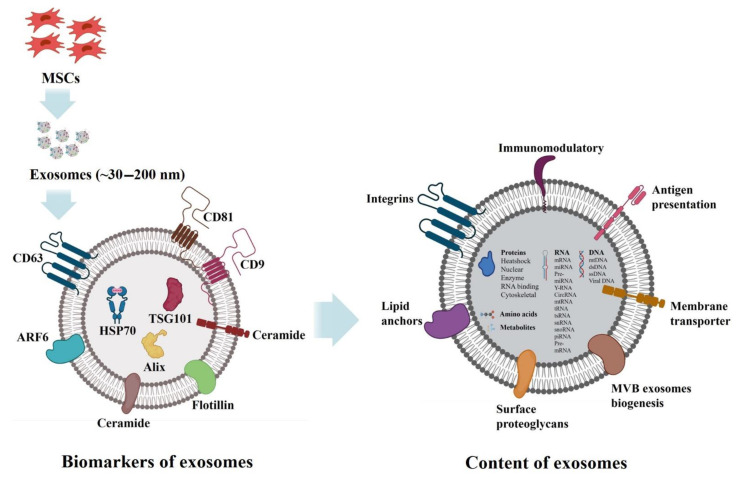
Typical exosomes. A powerful communication system between local and distant cells in the human body with pleiotropic functions. Exosomes are a type of extracellular vesicle secreted by cells such as MSCs that carry therapeutic payloads, including proteins, nucleic acids, lipids, enzymes, and metabolites.

**Figure 2:**
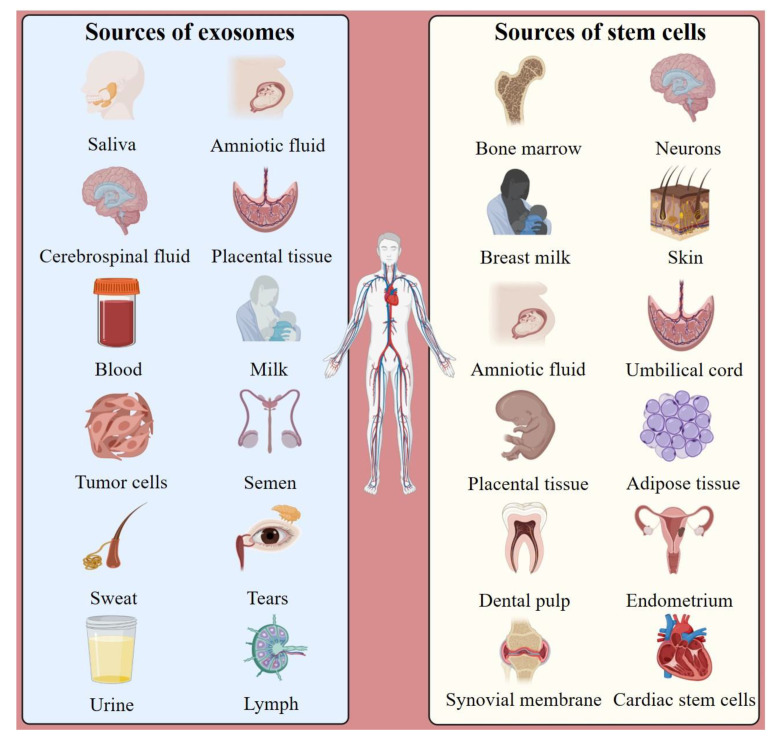
Sources of exosomes and stem cells. Exosomes have been detected in almost all body fluids, including, amniotic fluid, urine, cerebrospinal fluid, blood, serum, breast milk, malignant ascites, saliva, bile, etc. MSCs are non-hematopoietic, multipotent, adult stem cells which can be isolated from bone marrow, umbilical cord, placental tissue, adipose tissue, dental pulp, neurons, skin, breast milk, etc.

Exosomes are membranous extracellular vesicles that range from 30–200 nm in diameter. Exosomes have been found to be secreted by most cell types including immune cells (B cells, T cells, mast cells, dendritic cells), neuronal cells, epithelial cells, endothelial cells, embryonic cells, cancer cells, and mesenchymal stem cells (MSCs). The term “extracellular vesicle” broadly encompasses several types of vesicles including exosomes, microvesicles, and apoptotic bodies. However, the word “exosome” specifically denotes vesicles that are formed inside multivesicular bodies (MVBs) within cells [1,2,3]. Exosomes carry vital information and macromolecules from their source of origin and thus have a significant role in cell–cell communication. These macromolecules consist of a variety of proteins, enzymes, transcription factors, lipids, extracellular matrix proteins, receptors, and nucleic acids, and can be found both on the inside and outside of the exosomal surface (Figure 1). Exosomes have been detected in almost all body fluids in both heathy and disease conditions, including fluids such as urine, blood, serum, breast milk, amniotic fluid, cerebrospinal fluid, malignant ascites, saliva, bile, and lymph (Figure 2) [4,5,6,7,8,9,10,11,12].

Exosomes were first discovered by Pan and Johnstone while investigating the maturation mechanisms of sheep reticulocytes into erythrocytes [13,14,15]. The researchers discovered a type of vesicle (which they later named: “exosome”) that was released from reticulocytes and contained lipids, proteins, and enzymes of reticulocyte origin [16]. Exosomes were initially assumed to be cellular debris or garbage disposal and considered signs of cell death [17,18,19]. Since their discovery, extensive research has been carried out to determine the biology, function, and potential clinical uses of exosomes. It is now established that exosomes are released by donor cells into the extracellular environment to perform diverse biological functions, including intracellular communication and the exchange of genetic material and proteins between a parent cell and surrounding cells (Figure 1) [20,21]. The clinical importance of exosomes has been established in their use as alternatives to liposome-mediated drug delivery in cancer immunotherapy. Exosomes are also a promising biological gene delivery system due to their microRNA and mRNA content [22,23,24,25]. However, there are still many aspects of exosomes that are not fully understood or characterized. For example, as many potential targets for cancer therapy are tumor-specific biomarkers, it is crucial to study the biomarkers present on the surface of exosomes in order to develop tumor-targeting therapies [26,27]. The great potential of these small wonder vesicles to aid in gene delivery, disease diagnostics, intracellular communication, drug delivery, and biomarker-driven therapies has progressively drawn the attention of researchers.

**Figure 3:**
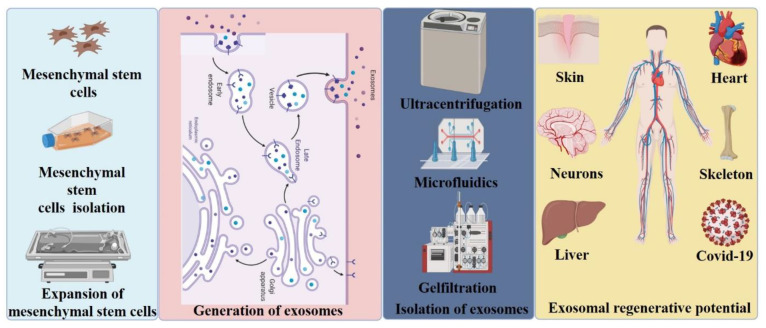
MSC-derived exosome methodology. Graphical representation of isolation of MSCs, exosome biogenesis, isolation of exosomes. and therapeutic applications of exosomes in regenerative medicine.

Stem cell therapies have increasingly gained momentum in treatment of disease; much of the research that has been done in recent years has focused on the potential significance of applying MSCs [28,29]. MSCs are non-hematopoietic, multipotent, adult stem cells which can be isolated from many biological sources including bone marrow, umbilical cord, adipose tissue, brain, spleen, kidney, and liver (Figure 2) [30,31,32,33,34,35]. MSCs can differentiate into adipocytes, chondrocytes and osteocytes, as well as endodermal (hepatocytes) and ectodermal lineages (neurocytes) [36]. In addition to their mechanical differentiation properties, MSCs also secrete exosomes and biomolecules including cytokines, chemokines, and growth factors. Though initial reports indicated that MSCs may play a critical role in tissue repair, investigations have shown poor survival and low grafting potential of MSCs in damaged tissue areas, limiting MSC effectiveness in tissue repair [37,38,39,40,41]. Further studies have demonstrated that beneficial effects of MSCs applications in repair are attributable to paracrine signaling, which includes secreted vesicles such as exosomes [42,43,44,45,46]. Interestingly, several studies indicate that exosomes secreted by MSCs can replace the MSC-based stem cell therapies in various injury and disease models [28,29]. For example, MSC-derived exosomes (MSC-Exos) have been shown to induce repair in mouse models of wound healing and myocardial infarction (Figure 3) [47,48,49,50,51]. In particular, investigations have revealed that exosomes secreted by placental umbilical cord MSCs play a significant role in wound healing and tissue regeneration [52]. Similarly, in the past few decades, studies have demonstrated that MSC-Exos can have advantageous effects in various contexts including neurological, respiratory, cartilage, kidney, cardiac, and liver diseases, bone repair, and cancer (Figure 3) [28,29,48,53,54,55,56,57,58,59]. 

In short, MSC-Exos can serve as a smart drug delivery approach through the transportation of exogenous chemicals and biomolecules for stem cell-free regenerative medicine. MSC-Exos have many potential therapeutic advantages when compared to synthetic nanoparticles, liposomes, single molecules, and cells. This stems from their novel beneficial characteristics such as smaller size, lower complexity, lack of nuclei (thus preventing neoplastic transformation), increased stability, easier production, longer preservation, and potential for loading proteins, small molecules, or RNAs for delivery of biomolecules [60]. MSC-Exos can also be modified to display distinct antibodies or surface receptors to transfer therapeutic payloads to specific organs, tissues, and cells. Additionally, MSC-Exos host numerous types of biological molecules, enabling them to participate in various therapeutic approaches simultaneously, which cannot be accomplished with conventional small molecules. Therefore, here, we review the recent advancements in the field of molecular mechanisms of exosomes in regenerative medicine and exosome research, as well as address the potential therapeutic approaches of exosomes in tissue regeneration due to disease and injury recovery. 

## 2. Exosome Biogenesis, Secretion and Uptake

**Figure 4:**
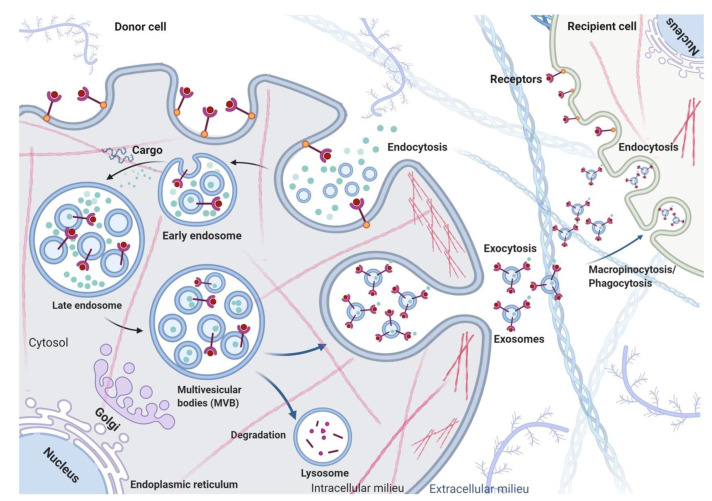
Exosome biogenesis, secretion and uptake. Extracellular milieu is composed of various components including proteins, lipids, small molecules, numerous metabolites, which enter the cell through the process of endocytosis. This process leads to the formation of early endosomes; cargo is wrapped into intraluminal vesicles within multivesicular bodies (MVB) upon inward budding of the membrane. Further, early endosomes are transformed into late endosomes, which later give rise to the multivesicular bodies. The modified multivesicular bodies have a cargo of extracellular milieu and cytoplasmic constitutes. Multivesicular bodies and their exosomal content can follow two primary pathways. In the first, they can be fused with autophagosomes and follow the degradation pathway through lysosomes. In the second pathway, multivesicular bodies can fuse to the plasma membrane through the microtubule and cytoskeletal network, and be released by budding from cytomembrane. Furthermore, the exosomes released through exocytosis interact with recipient cells through cell signaling molecules on their respective surfaces. Exosomes can also enter into recipient cells by employing different mechanisms such as endocytosis, macropinocytosis, phagocytosis and direct fusion of plasma membranes. Various proteins are involved in exosome biogenesis, secretion and uptake, include ESCRT, AAA ATPases, ESCRT-associated protein, SNAREs, Rabs, and other enzymes (Table 1).

The structure and composition of exosomes depends on several factors including the donor cell, microenvironment, and physiological conditions. Exosomes are formed from endosomal vesicles via the exocytosis process (Figure 4). As cargo transporters, exosomes can carry proteins, peptides, nucleic acids, and lipids. Studies investigating the protein composition of exosomes have shown that, while some proteins specifically arise from parental tissue, some were unique to exosomes [1,2,9]. Specific proteins contained within exosomes include those present in the endosome, plasma membrane, and cytoplasm, implying differential selection [20]. Additionally, several studies have shown that exosomes carry nucleic acids including different RNA types, e.g., microRNA, messenger RNA, and non-coding RNA [61,62,63,64,65,66,67]. Interestingly, the composition of proteins, peptides, and nucleic acids in exosomes are independent of donor cell types; on the other hand, the lipid composition in exosomes primarily depends on exosome-producing cells. Typically, exosomes contain plasma membrane lipids including sphingomyelin (SM), desaturated phosphatidylethanolamine, phosphatidylserine (PS), desaturated phosphatidylcholine (PC), cholesterol (CHOL), GM3, and ganglioside [68,69,70,71,72,73].

The biogenesis of exosomes occurs via exocytosis: a depiction of exosome biogenesis, secretion, and uptake is detailed in Figure 4. Exosomes are generated from multivesicular endosomes (MVEs); generally, the intraluminal vesicles (ILVs) of MVEs are subjected to lysosomal degradation by hydrolases. However, escaped MVEs, such as multivesicular bodies (MVBs), can directly fuse with cellular plasma membrane and, through the process of budding, are subsequently secreted to the extracellular milieu as exosomes. There, the exosomes have pleiotropic functions through paracrine signaling [20]. Currently, little is known about the underlying mechanism behind the sorting of exosomes into the different populations. Studies have revealed that MVBs specifically contain various lysosome-associated molecules such as lysosomal-associated membrane protein 1, 2, and 3 (LAMP-1, -2, -3), tetraspanins, and a cluster of differentiation factors (CD-107a, CD-107b, CD-208 or CD-63) (Figure 1), whereas late endosomes possess major histocompatibility complex (MHC) class II [20,74]. Hanson and Cashikar explored the morphogenesis mechanism of MVBs and found that the endosomal sorting complex required for transport (ESCRT) plays a crucial role in driving both exosomal and ectosomal biogenesis [75]. ESCRT comprises approximately 30 different proteins that are organized into four machinery complexes, namely ESCRT-0, -I, -II, and -III in association with vacuolar protein sorting associated protein 4 (VPS4), vesicle trafficking 1, and apoptosis-linked gene 2-interacting protein X (Alix) which is also called programmed cell death six interacting protein [75]. The initial ESCRT-0 complex assists in recognizing and sorting ubiquitinated intracellular cargos that are prescribed for lysosomal degradation. ESCRT-I and -II contribute to deforming the membrane into buds with sequestered vesicles, whereas ESCRT-III plays a role in vesicle scission [76]. ESCRT-independent biogenesis mechanisms are proposed to involve tetraspanins (CD63, CD9, CD37, CD82 or CD81), which have been identified as exosomal markers. These proteins are vital in extracellular vesicle biogenesis and essential for extracellular vesicle secretion and uptake by receptor cells. Hydrolysis of sphingomyelin into ceramide is also known to contribute to the biogenesis of exosomes [20,77]. As a summary, Table 1 includes the composition and functions of proteins, e.g., ESCRT, AAA ATPases, ESCRT-associated proteins, SNAREs, Rabs, and other enzymes, that are actively involved in exosome biogenesis, sorting, transport, and secretion [78,79,80,81,82,83,84,85,86,87,88,89].

## 3. Complicated Architecture of Exosomes

All exosomes share typical characteristic compositions of donor cells, and cargo can include proteins (tetraspanins, annexins, heat shock proteins, etc.), lipids (glycosphingolipids, sphingomyelins, cholesterol), genetic materials (DNA, tRNA, mRNA, miRNA, small and long noncoding RNAs (sncRNA and lncRNA, respectively)), and small-molecule metabolites (amino acids, ATP, amides, sugars, etc.) (Table 2, Table 3 and Table 4) [67,91,92,93,94,95,96,97,98]. The main database available for protein content in exosomes is ExoCarta (http://exocarta.ludwig.edu.au/ accessed on 17 April 2021); the recent version of ExoCarta includes listings from over 286 exosomal investigations annotated with International Society for Extracellular Vesicles’ minimal experimental requirements for definition of exosomes: data include 41,860 proteins, 1116 lipid molecules, and more than 7540 RNAs. Other databases include Vesiclepedia (http://www.microvesicles.org/ accessed on 17 April 2021), and the Urinary Exosomes Protein Database (http://dir.nhlbi.nih.gov/papers/lkem/exosome/ accessed on 17 April 2021) [99,100,101,102].

## 4. Proteins

Exosomal proteins perform various functions such as targeting/adhesion, anti-apoptosis, membrane fusion, signal transduction, metabolism, and structural dynamics [2,9]. There are similarities in protein content between species, as the available proteomic data for exosomes isolated from mouse and human dendritic cells (DCs) suggest that about 80% of the proteins are conserved in the two species [2,9,121]. However, based on proteomic studies, exosomes isolated from different cell types contain specific groups of proteins depending on the secreting cell types (Table 2). Western blot and fluorescence-activated cell sorting analysis can identify known cellular proteins in exosomes prepared from different cell types [74,107,108,135,136]. For unknown cellular proteins, mass spectrometry associated with trypsin digestions can analyze exosomes derived from cells like mast cells, DCs, and enterocytes [2,105,112,121,137,138,139,140]. These methods have been used to identify cytosolic proteins present in exosomes including actin, tubulin, cytoskeletal components, and actin-binding proteins, as well as Rab and annexins, which are important in intracellular membrane fusions and transport function (Table 2 and Table 3). Exosomes also contain different types of 14-3-3, heterotrimeric G proteins, and protein kinases, which are critical in signal transduction during critical physiological processes. Further, exosomes derived from human DCs and enterocytes contain various types of metabolic enzymes such as enolases, peroxidases, and lipid kinases. The two constitutive forms of heat shock proteins (i.e., HSP70 and HSP90) are found in exosomes which perform the function of antigen presentation and aid in loading antigenic peptides onto MHC class I molecules (Table 3). Notably, the MHC class I molecules are present in most isolated exosomes [141,142,143,144].

Exosomes also consist of proteins that are involved in specific cellular functions. For example, MHC class II molecules are present in large amounts in exosomes. Exosomes derived from DCs contain CD86, which acts as costimulatory molecule for T cells, and can contain cell-specific transmembrane proteins such as αM, β2 on DCs, and T cells, α4B1. Other exosome proteins include immunoglobulin-family members, intercellular adhesion molecule 1 (ICAM1)/CD54 on B cells and cell-surface peptidases such as dipeptidyl-peptidase IV/CD26 on enterocytes and aminopeptidase N/CD13 on mastocytes [145]. Additionally, exosomes harbor a vast variety of glycosylphosphatidylinositol (GPI) anchored proteins, nuclear proteins, and proteasome related proteins. Finally, the proteome of exosomes of various cell types are also divided into several signaling molecules and enzymes complexes (Table 2 and Table 3) [122,123,124,125,146].

## 5. Lipids

Lipids are the least studied but most crucial components within exosomal membranes. Exosomes contain an abundance of lipids including glycosphingolipids, sphingomyelins (SM), cholesterol (CHOL), and phosphatidylserine (PS). Lipids not only play an important role in the structure of exosomal membranes, but also facilitate the formation of exosomes and their release into extracellular milieu [73,147,148]. Exosomes mainly contain monounsaturated fatty acids, polyunsaturated fatty acids, and saturated fatty acids, though the lipid composition of an exosome depends on the parent cells from which the exosome is derived [69,70,71,72]. Interestingly, exosomes can transport several bioactive lipids and lipid metabolism-related enzymes [126,149]. Fatty acids such as arachidonic acid (AA), leukotrienes prostaglandins, phosphatidic acid, docosahexaenoic acid (DHA), and lysophosphatidylcholine (LPC) have been found in MSC-Exos. The lipid metabolism enzymes of exosomes can modulate the cell homeostasis of a beneficiary [71,150].

The lipid classes found in different exosome types based on the published investigations are shown in Table 4. Methods such as thin-layer chromatography, gas–liquid chromatography, and mass spectrometry have been used to identify the lipids in exosomes [71]. Studies have shown that the content of CHOL, SM, glycosphingolipids, and PS is 2–3 times higher in exosomes compared to their parent cell. In addition to this, the majority of exosomes show lower content of PC and phosphatidylinositol (PI) than in parent cells. Additionally, lipid content can significantly vary by cell type. The lipid composition of exosomes isolated from hepatocyte cells (HepG2/C3a Oli-neu cells), prostate cancer cells (PC-3 cells), and PC-3 cells+ hexadecylglycerol (HG: a precursor of ether phospholipids) exhibit some similarities [66]. Furthermore, the exosome preparation from Oli-neu cells has lower enrichment of SM and higher enrichment of CHOL, as compared to exosomes isolated from PC-3 cells [66]. Reticulocyte-derived exosomes present high levels of CHOL as compared to other cells [56], while the enrichment of exosomes from adipocytes was found to contain a higher level of SM and lower level of PS than other various preparations in Table 4 [134].

Though lipidomic studies conducted on exosomes isolated from different cell types have been published, results are typically given only in the form of the number of different lipid classes. For example, the lipidomic study of exosomes isolated from colorectal cancer cells (LIM1215) identified a total of 500 lipid species [151], whereas *Brzozowski* et al. reported total of 187 lipid species identified in exosomes enriched from prostate cancer cell lines, i.e., PC-3, RWPE1, and NB26 [71,72,128]. Further studies have shown that exosomes secreted from epithelial cells (RWPE1) contain high amounts of fatty acids, prenol, and glycerolipids lipids. Exosomes from prostate cancer cell lines (PC-3 and NB26) show an abundant amount of sterol lipids, glycerophospholipids, and sphingolipids. In a lipidomic analysis of exosomes isolated from U87 (glioblastoma cells), Huh7 (hepatocellular carcinoma cells), and human bone marrow derived MSCs, *Haraszti* et al. showed that the lipid composition of MSCs and Huh7 exosomes were similar to each other but distinct from the U87 exosomes [152]. 

## 6. RNAs

Another important cargo of exosomes is RNAs, leading to the emergence of exosomes as a mediator of intracellular communication and a component of various signaling pathways [153,154,155]. MSC-Exos include RNA, which was found to be enclosed within cholesterol-rich phospholipid. This was demonstrated by the RNA cargo’s vulnerability to RNase degradation only in the presence of sodium dodecyl sulfate (SDS)-based lysis buffer, a chelator of cholesterol, cyclodextrin, and phospholipase A2 [154,155]. 

Ethidium bromide staining, a technique routinely used for the detection of RNA, of MSC exosomal RNAs found that they consist of mainly short RNAs (<300 nt), whereas 28S and 18S RNAs were not visible [67,154,156,157]. A primary exosomal mechanism of action is thought to be post-transcriptional gene regulation via microRNA content (miRNAs, miRs), which are small, endogenous RNA molecules around 22 nucleotides in length. miRNAs have been shown to play pivotal roles in health and disease, including cancer, cardiovascular diseases, and wound healing [158]. Microarray hybridization of MSC-derived exosomal RNA against probes for 151 miRNAs revealed the existence of 60 miRNAs and ribosomal RNA degradative products [154,155]. Comparative analysis of the composition of MSC exosomal miRNAs with their cellular miRNA revealed that 106 miRNAs from the MSCs were not secreted in the MSC exosomes. These results suggested that MSCs secrete a select population of miRNA through a regulated process. Furthermore, an ample amount of passenger miRNA has been found in MSC-Exos [159].

There have been several studies conducted on exosomal miRNAs involved in intracellular communications and disease [61,62,64,65,66]. The plasma-derived exosomal miR-92a showed an anti-apoptotic effect on fibroblast-like synoviocytes, which ultimately leads into the destruction of bone in rheumatoid arthritis patients [66]. Researchers have found that MSC-derived exosomal miRNAs can both promote [160] and reduce tumor growth [161]. For example, *Lee* et al. found that miR-16, a microRNA known to target vascular endothelial growth factor (VEGF), was abundantly present in MSC exosomes, leading to an antiangiogenic effect on tumor cells [161]. MSC-derived exosomal miRNAs also play an essential role in cardiovascular protection and repair by regeneration, as well as inhibition of cardiac apoptosis and fibrosis [162]. *Shao* et al. discovered that MSC-Exos enclosed a higher amount of cardioprotective miRNA such as miR-29 and miR-24, and lower amount of cardiac-offensive miR-21 and miR-15 as compared to MSCs [163]. Further, it was found that human amniotic epithelial cell-derived exosomal miRNAs play a crucial role in wound healing by promoting cell migration and proliferation of fibroblasts [164]. Human amnion MSC-derived exosomal miRNA, miR-135a, promotes wound healing and fibroblast migration by downregulating large tumor suppressor kinase 2 expression [165]. *Wu* el al. further found that MSC-derived exosomal miR-100 provides protection to the articular cartilage and helps in regulation of cartilage homeostasis in the OA mice model via inhibition of mTOR-autophagy pathway [149]. Further, it has been identified that plasma-derived exosomal miRNAs are involved in ‘extracellular matrix-receptor interaction’ and contribute to Hirschsprung’s disease through interfering in cell junctions [166]. Human adipose stem cell-derived exosomes loaded with miR-21 mimics play a critical role in cell proliferation and migration of keratinocytes, and treatment of diabetic chronic wounds with miR-21 mimics results in accelerated healing by collagen remodeling, increasing re-epithelization, vessel maturation, and angiogenesis in vivo [167]. Further descriptions of the role of exosomal miRNA are discussed in later sections.

## 7. Emerging Technologies for Exosome Isolation

The majority of the cells in the body secrete exosomes in the extracellular milieu; however, exosomes are also found with other body fluids (Figure 2). Since their discovery, several methods have been developed to isolate the exosomes from body fluids. In the past decades, there have been many advances in exosome detection and separation techniques, resulting in higher recovery, purity, sensitivity, and specificity of isolated exosomes (Table 5). Still, due to overlapping size range, small sizes, and similar morphologies to other extracellular vesicles challenges persist in isolation methods. 

The most common and traditional method of exosome isolation is ultracentrifugation/differential ultracentrifugation, which separates exosomes based on size and density [170,171]. This technique is comparatively cost-effective and secure, though time consuming. It is generally used for the isolation of exosomes from large volumes of biological cultures. The main disadvantage of this method is a lack of specificity, in that the separated exosomes could contain other extracellular vesicles of similar sizes. To overcome this problem, it is recommended to use iodixanol or sucrose cushions in addition to differential ultracentrifugation [172,173,174]. 

Ultrafiltration is another conventional method used for the isolation of exosomes. In this method, exosomes can be isolated based on their molecular weight or size. For example, exosomes can be separated using defined molecular weight cut-off membrane filters. The filtration method is much faster than differential ultracentrifugation and does not require any kind of unique instrument [168,171,175,176,177]. However, a major drawback is a lack of purity in the isolated fraction. Similar to ultracentrifugation, it is hard to omit compounds of other molecules with similar sizes to exosomes [178,179].

In addition to ultrafiltration, size exclusion chromatography (SEC) also separates exosomes on the basis of size or molecular weight [180,181]. SEC isolates exosomes with high purity and high yield, and acts as an essential tool in the process of exosome purification. In SEC, a column made with a solid-phase matrix of beads, with pores of different sizes, is used to separate macromolecules and other particulate matter [47,182]. SEC can be used in combination with ultracentrifugation or other techniques for higher yield of exosomes [183,184]. 

An immunoaffinity chromatography method can be used to enhance the purity of separated exosomes. Exosomal membrane proteins and receptor molecules are used to develop this highly specific method. In this technique, the exosomes can be captured on the column by immunoaffinitive proteins and their specific antibodies [185,186,187]. The immunoaffinity chromatography technique is appropriate for smaller-scale production of exosomes from fewer sample volumes. A microplate-based enzyme-linked immunosorbent assay (ELISA) is the best example of immunoaffinity-based chromatography approach used for quantifying the captured exosomes from biological samples such as a serum, plasma, and urine [188].

Exosome purification can also be carried out by precipitation [189,190]. Precipitation-based exosome isolation is used to concentrate the exosomes from biological fluids. Exosomes can be precipitated from cell culture media by altering their dispersibility and solubility. This can be achieved by commercially available precipitation reagents such as polyethylene glycol (PEG) [177,191]. Currently, numerous precipitation-based exosome isolation kits are available in the market. These are compatible with biological fluids such as urine, plasma, serum, cerebrospinal fluid, and cell culture medium [192,193].

Recently, microfluidic-based methods were developed for the rapid and efficient isolation of exosomes from biological samples. The main advantages of these techniques are remarkable reductions in reagent consumption, sample volume, analysis cost, and isolation time [194,195,196]. As scalability is enhanced through technique modification and available technologies, the needs of health care applications such as reproducibility, reliability, low cost, and speed can eventually be fulfilled. A full summary of advantages and disadvantages of the exosome isolation methods is given in Table 5.

## 8. Applications of Exosomes in Injury and Disease

Exosomes, and specifically exosomes derived from mesenchymal stem cells, have been found to have enormous benefits in a variety of diseases and injuries through the proteins and RNAs that they contain. Additionally, because exosomes are representations of their parent cells, as the cellular environment changes, so exosomes change. As such, the number and content of exosomes can be used as a biomarker for changing conditions in disease. In the following sections we summarize recent findings in problems such as wound healing, neurological damage, and hepatic diseases.

## 9. Wound Healing

**Figure 5:**
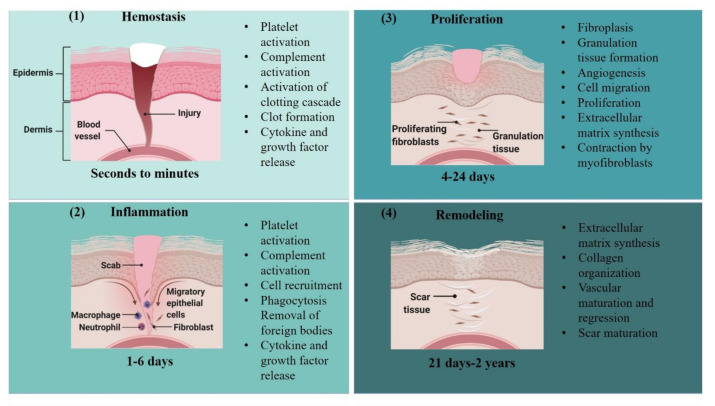
Phases of wound healing. The main events implicated during wound healing are hemostasis, inflammation, proliferation, and remodeling. (1) Hemostasis is the process of blood clotting and is the shortest phase in wound healing, lasting for only a few minutes. (2) The inflammatory phase comprises of blood coagulation, phagocytosis, removal of foreign bodies, and recruitment of growth factors and anti-inflammatory cells at the site of injury. (3) The proliferation phase encompasses fibroplasia, angiogenesis and cell migration, cell recruitment, re-epithelialization, and wound contraction. (4) Finally, in the remodeling phase, type I collagen replaces type III collagen in the wound site. Eventually, scar formation occurs through apoptosis.

Skin damage can commonly arise due to factors such as the sun, parasites, or a fall, often leading to open abrasions with potential for infection. Injuries to the skin are healed in an intricate process that takes place in four overlapping stages: (1) hemostasis, (2) inflammation, (3) proliferation; and (4) maturation/remodeling (Figure 5) [197,198,199]. In the first stage, hemostasis, platelets form a blood clot to prevent blood loss. Simultaneously, the platelets secrete hormones, cytokines, and chemokines, including tumor growth factor-β (TGF-β), epidermal growth factor (EGF), platelet-derived growth factor (PDGF), and fibroblast growth factor (FGF), to attract inflammatory cells (growth factors important in wound healing are summarized in Table 6) [9,40,200,201,202,203,204]. Inflammation, the second step of wound healing, begins within 24 h of injury as neutrophils infiltrate the wound and secrete products, such as toll-like receptors (TLRs) and nuclear factor к-light-chain-enhancer of activated B cells (NF-кB), to attract and activate pro-inflammatory (M1) macrophages [9,40,201,202,204]. M1 macrophages phagocytose pathogens, produce an oxidative burst, and remove apoptotic cells before products including signal transducer and activator of transcription 3 (STAT-3) promote the polarization of M1 macrophages into anti-inflammatory M2 macrophages, thus stimulating inflammatory resolution [205,206,207,208]. Proliferation then begins as keratinocytes and fibroblasts proliferate at the edge of the wound. Increased levels of VEGF and FGF stimulate angiogenesis, the process by which new blood vessels are formed to transport necessary nutrients, oxygen, and growth factors to the damaged tissues. Fibroblasts secrete immature type III collagen to form a new extracellular matrix (ECM), and then differentiate into myofibroblasts. These cells have contractile abilities, pulling together the edges of the wound [197,198,203,206,209]. Finally, during the maturation phase, the former ECM gets degraded by a variety of enzymes, including matrix metalloproteinases and plasminogen activators, as the type III collagen is substituted by mature type I collagen. The remodeling of the scar is a longer process than other stages of wound healing: over months or years, the scar tissue reaches its final appearance [198,209]. The proper sequence, timing, and regulation of these stages are critical during wound healing; any delinquency in this progression can result in the formation of chronic ulcers or hypertrophic scarring [197,199,210]. The major risk factors in this are underlying conditions such as aging, diabetes, and recalcitrant infections. Intemperate fibroblast activity results in hypertrophic scarring and may degenerate into keloids [210,211,212].

One of the promising approaches for this is an exploitation of cellular therapies using MSCs and the possible role of MSC-Exos in wound healing and regeneration [39]. Though studies using MSCs have revealed that both autologous and allogenous MSCs give promising results [263], several studies have demonstrated that the effectiveness and the regenerative capacity of the conditioned media from MSCs is similar or greater than MSCs when applied to chronic wounds [37,39,40,41,48,264]. As such, it has been found that the MSC-derived secretome, in the form of exosomes, carries soluble factors and metabolites that play an important part during wound healing [40,41,265]. Recent research has highlighted the potential of MSC-Exos, and in particular the growth factor and microRNA content of exosomes, as a therapeutic treatment of chronic skin ulcers and hypertrophic scarring, as well as the possible role of exosomes in the modulation of different stages of wound healing (see Table 7 for an expansive list of microRNAs in wound healing) [48,198,200,217,266,267]. Exosomes can target several pathways including phosphoinositide 3-kinase (PI3K)/AKT, ERK, and STAT-3 which are vital in facilitating and accelerating wound healing through downstream targets such as hepatocyte growth factor (HGF), insulin-like growth factor-1, nerve growth factor (NGF), and stromal cell-derived factor [48,50,198,268,269,270,271,272,273]. Further, a study by Shabbir and team demonstrated a trending increase in VEGF induced by MSC-Exo administration [48]. In addition to activation of growth factors through downstream processes, growth factors such as VEGF, HGF, and PDGF have been found in exosomes isolated from MSCs of different sources [274,275]. TGF-β has been found in low levels in umbilical cord MSC-Exos [274,276], and when exosomes are further loaded with TGF-β cargo, can stimulate vascularization and matrix remodeling [277]. Exosomes, by proxy of their effect on and containment of growth factor, possess cellular proliferation and differentiation modulating properties along with high immunomodulating, immunosuppressing, and angiogenic activities, which have been demonstrated both in cell culture and animal models [50,266,269,278]. 

Exosomes have been identified as an important regulator of inflammation. Importantly, exosomes are even beneficial in instances of chronic inflammation, such as found in diabetic patients, where higher levels of glucose thwart the proper macrophage polarization (M1 → M2), making these patients prone to chronic skin wounds [40,201,202,203,204,205]. MSC-Exos can promote macrophage polarization and attenuate cytokine secretion to resolve the inflammatory stage [49,201,202]. These effects can be explained in part through microRNA, as microRNAs such as miR-132 are highly upregulated during inflammation and can induce M2 polarization through regulation of TLRs [285,286,287]. Studies have found that miR-132 located within MSC-Exos can elevate IL-10 expression and decrease levels of NF-кB, IL-6 and IL-1β in favor of inflammation resolution [288,289,290]. Broadly, exosomes have shown significant beneficial effects on proliferation, collagen deposition, and angiogenesis, even in states of chronic wounds and comorbidities such as diabetes [272,273,291]. Several microRNAs participate in this step including miR-132, miR-126, and miR-21. miR-132 plays a role in proliferation, as it can increase the activity of the STAT-3 and ERK pathways, thereby promoting keratinocyte growth. Exosomes loaded with miR-132 improved angiogenesis by increasing the tube formation of endothelial cells [211,289]. In a hypoxia-like environment, exosomes have been found to be abundant with miR-126, which aids in angiogenesis through downstream activation of PI3K/AKT signaling [292,293]. Though many miRNAs can be found in exosomes, some are highly expressed, including miR-21 [280]. This important microRNA has been shown to be impactful in several diseases and injuries. miR-21 promotes migration of keratinocytes and fibroblasts, stimulating re-epithelialization, and promotes collagen synthesis [280,285,294,295,296]. Further, miR-21 can resolve inflammation, as miR-21 is increased in macrophages after they envelop apoptotic neutrophils, a key process in the transition from inflammation into proliferation stages [39,263]. Additionally, miR-21 can downregulate TLR-4-mediated inflammation through the inhibition of the expression of programmed cell death protein 4 [39,281]. miR29a and miR29b are also shown to be have elevated levels in MSC-Exos and play pivotal roles in regulation of TGF-β and cell growth; in addition to the highly expressed miR-23a, the 29 family also has a large impact on angiogenesis [280,284]. 

Exosomes, and MSC-Exos specifically, show great potential for the promotion of rapid and efficient wound healing. Exosomes can be applied directly to an injury, which in animal models has been shown to promote collagen synthesis and the proliferation and migration of fibroblasts and keratinocytes (Table 8) [268,297,298]. These effects are shown to be due in part to exosomal regulations of microRNA levels and protease activities [297,299]. However, progressively more creative ways to utilize these versatile particles in novel technologies are being developed, such as the incorporation of exosomes into a gel, which is then applied to an injury. This treatment method has been shown to be more beneficial than a single, direct administration of exosomes due to a slow, steady rate of delivery, with some gels delivering exosomes for up to a week [300,301,302]. These scaffolds offer structure, hydration, and increased flexibility in treatment options, as hydrogels can been used for wounds from basic skin injuries to deep nerve damage [302,303]. In the continued development of wound healing strategies continue, the broad applicability and therapeutic benefits of exosomes will only expand the prospects and efficiency of wound healing technologies.

## 10. Brain Injury

**Figure 6:**
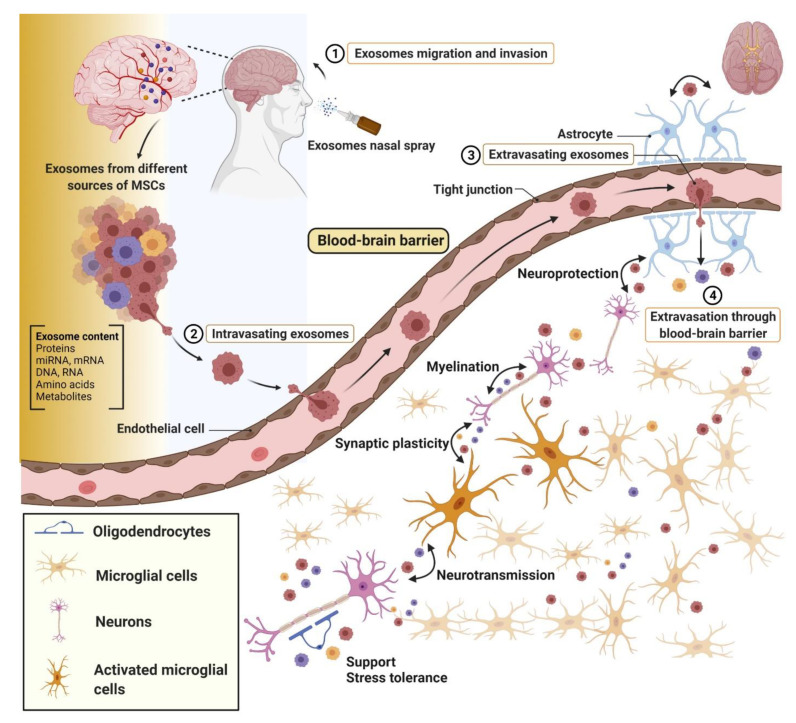
Schematic representation of functions of exosomes in neural cell communication. Exosomes released from different types of MSCs such as human umbilical cord, adipose tissue, bone marrow, and neural stem cells perform several investigated and suggested biological functions. Exosomes delivered via intranasal, intravenous, or other routes can migrate to the brain and penetrate the blood brain barrier. From there, the exosomes can enter general circulation and arrive at far off targets. Conversely, exosomes can travel across the blood brain barrier from inside the blood vessel into the central nervous system and be taken up by neurons and glial cells. Exosomes contain diverse contents (as depicted in Figure 1), that can influence inflammation, misfolded proteins, damage, and disease. In injury states such as TBI or stroke, exosomes interact with synaptic activity and neural survival, facilitating neurite outgrowth, and can promoting myelination and blood brain barrier repair.

Injuries to the brain, including traumatic brain injury (TBI) or stroke, can lead to long-term disability and decreased life expectancy, making them major health and economic issues [46,308,309]. These traumas can prompt rapid acute and long-term damage to neuronal tissue and function. Successful treatment of brain injuries is limited due to the need for swift diagnosis and difficulties in delivering therapeutics past the blood-brain barrier (BBB). Additional complications arise due to the myriad of changes that take place following brain damages. MSC-Exos are not only capable of crossing the BBB through intravenous or intranasal delivery, but also have beneficial effects in treating chronic inflammation in and promoting healthy healing, making them a potential therapeutic for complex brain injuries (Figure 6) [310,311,312,313]. 

Impacts from sports, car crashes, military experiences, or falls can lead to damage to the brain called a concussion, or traumatic brain injury (TBI). Originally thought to be an acute event, research has now shown that TBIs can lead to long-lasting effects on brain function, reducing life expectancy [308,309,314]. TBIs produce immediate trauma to the brain in which neurons, glia, and blood vessels stretch or tear, inducing apoptosis and damaging the BBB. This is followed by a pro-inflammatory immune response that recruits glial cells to the injury site; upon arrival to the injury, immune cells become activated, phagocytose dead and damaged cells, and secrete pro-inflammatory signals. In moderate to severe injuries, this inflammatory immune response does not properly resolve, causing chronic inflammation: complications of chronic inflammation can last weeks to years following injury [46,308,314]. MSC-Exos from various sources including bone marrow, umbilical cord, and adipose tissue have shown great potential in modulating the inflammatory response that follows a TBI [46,51,315]. When MSC-Exos were delivered 24 hours after injury in a model of TBI, controlled cortical impact, researchers found a decreased inflammatory response, which led to improved recovery via enhanced neurogenesis and angiogenesis [51,309,315]. This effect is due in part to microRNAs contained within exosomes, such as miR-9, miR-124, and miR-125b. These microRNAs regulate important cytokines such as IL-1β, and thus promote neurogenesis. Studies have found that treatment with exosomes can decrease inflammatory markers in a dose-dependent fashion [46,308,316,317]. Additionally, exosomes can regulate TLR-4 and macrophage polarization, thereby promoting recovery following a TBI [46,136,314,317,318]. Importantly, administration of MSC-Exos following a concussion can not only help the molecular changes that take place, but also lead to improvements in motor and cognitive deficits that commonly occur after brain damage [51,309,315,317,318,319,320].

Another common injury to the brain is a stroke, which is a major cause of morbidity globally, with 15 million estimated strokes every year worldwide [310,313,321,322]. There are different types of strokes which can be difficult to differentiate between creating complications in diagnoses, including ischemic (development of a clot that blocks blood flow to a part of the brain) and hemorrhagic (a blood vessel ruptures) [74,323,324]. Following a stroke, there is a loss of oxygen to the brain, cell death, and excess inflammation. Interestingly, exosomes are being considered as a potential biomarker for stroke severity, and importantly for diagnosis, stroke type [8,322,325]. *Kalani* et al. found that the microRNA content in secreted exosomes is contingent on the type of stroke a patient suffered from, with miRs such as miR-21-3p, miR-27b-3p, and miR-132-3p elevated in patients with an ischemic stroke [8]. In addition to exosomes serving as a stroke biomarker, MSC-Exos have highly beneficial properties in the treatment of stroke. The primary standard of ischemic stroke care is the delivery of tissue plasminogen activator (tPA); interestingly, the addition of exosomes to tPA treatment significantly improved functional outcome following stroke compared to tPA treatment alone [313,317]. Delivery of MSC-Exos in stroke models leads to long-term neuroprotection, improved neurogenesis and neurovascular remodeling, as well as enhanced behavioral and neurological performances in motor function, coordination, sensorimotor, and spatial learning [136,317,324,326,327,328,329,330]. Varied contents of exosomes have been shown to aid recuperation, from growth factors such as VEGF to microRNAs [46,331]. *Zheng* et al. identified that miR-25 in MSC-Exos improved cell viability following stroke through modulation of BCL2/adenovirus E1B 19 kDa protein-interacting protein 3, while in a model of middle cerebral artery occlusion, miR-133b secreted from MSCs led to improved neurogenesis and stroke recovery [46,310,311,330]. Similar to studies in TBI, MSC-Exos can improve recovery through modulation of inflammation [43,136,310,313,328]. A study by *Zhao* et al. found that delivery of MSC exosomes significantly decreased inflammatory signaling and promoted the polarization of microglia from M1 to M2 activation [136]. miR-21, miR-199a, miR-124a, and miR-17 are a few of many exosomal microRNAs that have been shown to play beneficial roles in neuroprotection, immune regulation, and rejuvenation after injury [324,332]. Together, these studies demonstrate that exosomes are highly beneficial for neurological injuries, not only due to the vast therapeutic value provided by exosomal contents, but also because these vesicles can bypass the BBB through both intravenous and intranasal administration. See Figure 6 for depiction of exosomal role in brain healing.

## 11. Hepatic Diseases

Liver diseases include illnesses such as cirrhosis (the scarring of the liver) and hepatocellular carcinoma, and worldwide account for approximately 2 million deaths annually [333]. Treatments for liver diseases vary, but several studies related to acute liver injury and other hepatic diseases have identified that exosomes may have dual function of therapeutic agents as well as specific biomarkers for liver disease diagnosis [334,335,336,337,338]. Recently, a report from *Momen-Heravi* and group has demonstrated that in alcoholic hepatitis patients, the number of exosomes was found to be elevated compared to the healthy population [338]. Certain RNAs are differentially affected in exosomes derived from patients with liver diseases, with RNAs such as miR-21 elevated in exosomes of patients with hepatocellular carcinoma [334,335,338,339]. These findings set a stage for exosomes as biomarkers for noninvasive detection of hepatocellular carcinoma and other acute liver diseases [340,341,342]. 

The regenerative capacities of exosomes in liver have been explored as therapeutic agents, as exosomes carry cargo over large distances for cellular communication. Several studies have found that treatment with exosomes benefits liver repair and regeneration following hepatic failure, an effect thought to be due to the promotion of angiogenesis via the Wnt signaling pathway [343]. Further, hepatocyte-derived exosomes can deliver the synthetic machinery to form sphingosine-1-phosphate in target hepatocytes, enabling cell proliferation and liver regeneration after ischemia, reperfusion injuries, or after partial hepatectomy [344]. It has also demonstrated that hepatocyte-derived exosomes stimulate hepatocyte proliferation in vitro and promote liver regeneration in vivo during acute liver injury; the underlying mechanism is thought to involve exosome-mediated transfer of neutral ceramidase and sphingosine kinase 2 at the site of regeneration. Additionally, it was revealed that after liver injury, the enhanced levels of circulating exosomes have proliferative effects [344]. The regenerative potential of MSC-Exos in carbon tetrachloride (CCl4)-induced liver injury has also been investigated. It was found that these exosomes effectively attenuated the CCl4-induced liver injury by promoting proliferative and regenerative responses [345,346]. The potential effect of exosomes derived from human-induced pluripotent stem cell-derived mesenchymal stromal cells was studied in hepatic ischemia-reperfusion injury. The administration of such exosomes showed promising effects in the recovery of hepatic ischemia-reperfusion injury, with suppressed inflammatory responses, attenuated oxidative stress responses, and inhibited cellular apoptosis, pointing to exosomes as a viable therapeutic option for liver diseases [347].

## 12. Cardiovascular Diseases

**Figure 7:**
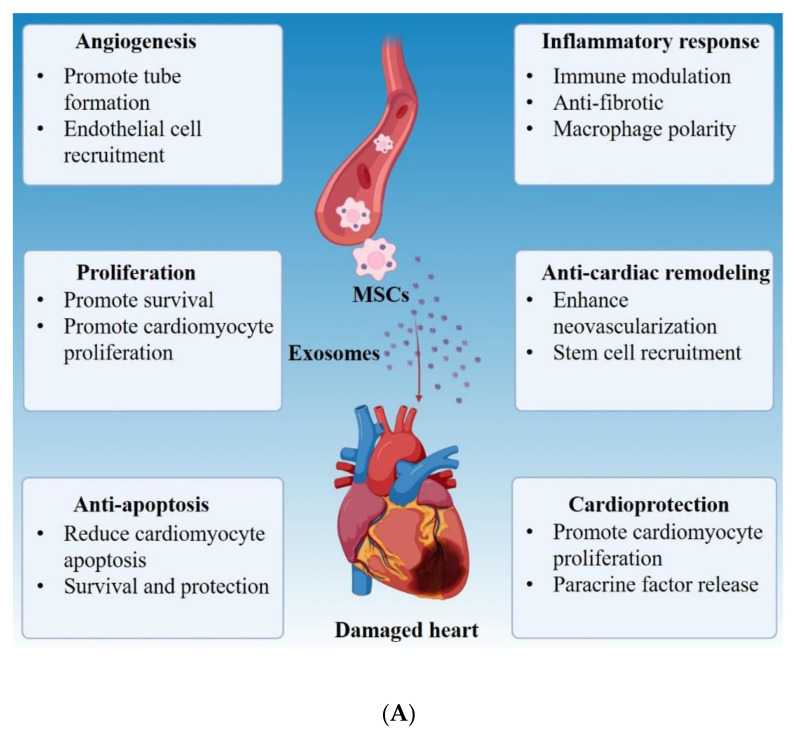

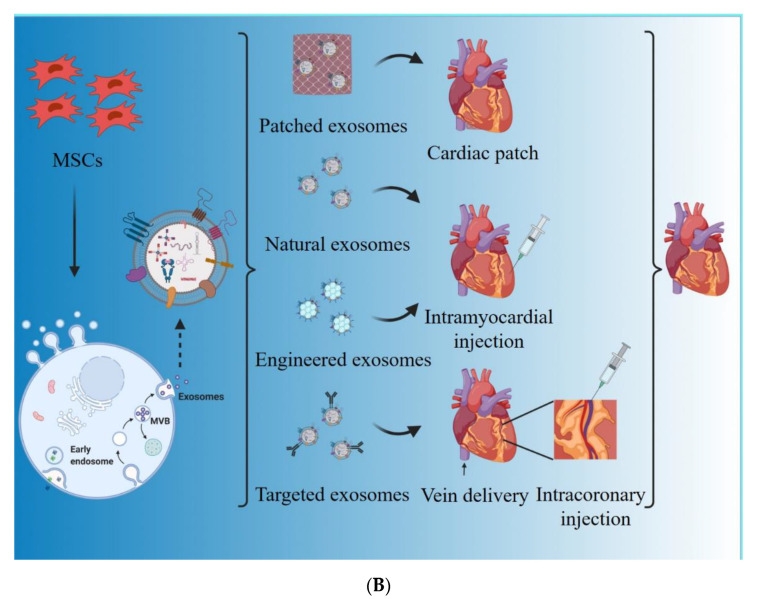
Stem cell-derived exosomes for cardiac repair therapies. (**A**) Exosomes isolated from different types of mesenchymal stem cells carry and deliver proteins, nucleic acids (DNA, miRNAs, mRNAs, and other RNAs) and metabolites to the damage heart tissue, consequently promoting cardioprotective effects. (**B**) Schematic representation of tissue engineering approaches in exosome-based cardiac repair therapies. MSCs or exosomes can be genetically modified with tRNA, miRNA or mRNA to express the desired gene using gene delivery methods or CRISPR/Cas9. MSC-derived exosomes can act as therapeutic vehicles to deliver biological molecules or drug molecules and further immersed in scaffolds and delivered as patch, injectable scaffold, or 3D tissue construct to increase the functions of exosomes. Similarly, exosomes can be chemically conjugated with targeted peptides to further enhance efficacy and retention when delivered intravenously.

Cardiovascular diseases (CVDs), such as heart failure and coronary artery disease, are some of the prime causes of morbidities and mortalities in the United States, accounting for a total of around 655,000 attributable fatalities per year and are associated with immense health and financial expenditure [348,349]. Conventional CVD therapies primarily consist of transplantations and therapeutics; however, receiving a transplant can be a very drawn-out process, and therapeutics have limited clinical efficacy. Therefore, focus has shifted to the development and validation of new therapeutics, with numerous cell-based therapeutic interventions initiated for the treatment of CVDs. Though cell-based treatment methods are promising, they face challenges such as low engraftment, poor survival rate of transplant cells, tumorigenesis potential, and immune rejection. Intriguingly, recent experimental data have suggested that myocardial protective functions through autocrine and/or paracrine actions of cell-based therapies may be achieved via through exosomes (Figure 7) [350,351]. Exosomes have major roles physiological and pathological cardiovascular processes including regulation of angiogenesis, cardiomyocyte hypertrophy, cardiac fibrosis, blood pressure control, and anti-apoptotic effect (survival) have been broadly acknowledged. Additionally, in the heart, cells including cardiomyocytes, cardiac fibroblasts, endothelial, vascular, cardiac progenitors, and stem cells release exosomes [352,353,354,355,356,357,358,359,360]. Moreover, exosomes lack some of the issues of cell-based therapies due to their low immunogenicity, minimal embolism risk, and biocompatibility, and can be delivered to the heart in a variety of ways, including engineered exosomes, endogenous exosomes, targeted exosomes, or exosomes contained in a patch [361,362]. The heart undergoes extensive cardiac remodeling following cardiac stressors such as myocardial infarction (MI)**,** to restore contractile function. Numerous studies have indicated that after cardiac stressors, endogenous exosomes can ameliorate heart function (Figure 7) [363,364,365]. This demonstrates that exosomes can have effective therapeutic utility in the treatment of CVDs.

The therapeutic potential of exosome-based cell-free therapy for CVDs applies to several diseases including MI, atherosclerosis, and dilated cardiomyopathy. The cardioprotective effect of exosomes can be augmented by pretreatments like hypoxia preconditioning, gene programming, or drug intervention [294,366,367]. In MI increasing evidence has shown that the administration of exosomes can enhance cardiac repair. For example, a study has revealed that MSC-Exos improve cardiac function by down-regulating the expression of CD68 [163]. Exosomes obtained from miR-146a-modified adipose-derived stem cells (ADSCExos) have been shown to attenuates acute MI-induced myocardial damage by suppressing the local inflammatory response through inhibition of the release of proinflammatory cytokines (IL-6, IL-1β, and TNF-α). The same report further demonstrated that ADSCExos improves cardiac functions by arresting cardiomyocyte apoptosis via early growth response factor 1 downregulation [368]. *Huang* and coworkers have discovered that exosomes from atorvastatin (ATV)-pretreated MSC (MSCATV-Exos) ameliorated cardiac dysfunction and reduced infarct area by diminishing IL-6 and TNF-α levels, promoting angiogenesis, and preventing apoptosis following MI. MSCATV-Exos are abundant in lncRNA H19 that regulates miR-675 expression and activation of pro-angiogenic factors [366]. Adipose-derived stromal cells (ADSC)-derived miR-93-5p-containing exosomes are also beneficial in the treatment of MI by conferring protection against autophagy, apoptosis, and inflammation [369]. Separately, in a mouse model of MI, hypoxia-derived exosomal miR125b-5p exerts cardioprotective function and enhances cardiac repair by suppressing the expression of pro-apoptotic genes *p53* and *BAK1*, thus inhibiting cardiomyocyte apoptosis [370]. 

The therapeutic promise of exosomes has also been investigated in the treatment of atherosclerosis. MSC-Exos serve a protective role in hindering the progression of atherosclerosis by inducing M1→M2 macrophage polarization through up-regulation of miR-let7 [371], an effect also displayed in different animal models of ischemia-reperfusion (I/R) injury [372,373]. Post myocardial I/R injury, exosomes aid in cardiac repair and contract myocardial infarct size by limiting cardiac fibroblast proliferation, creating an anti-inflammation microenvironment, and improving cardiac function mainly via the shuttling of miRNAs (miR-182, miR-146a, miR-181b, and miR-126) [372,373]. In addition, *Lankford* and colleagues have shown that the MSC-Exos are effective in dilated cardiomyopathy, as exosomes reduce ventricular dilation by hampering inflammatory cytokines expression and enhancing the production of anti-inflammatory M2 macrophages over M1 macrophages [374]. In chronic heart failure, a study has shown significantly elevated exosomal miR-146 levels, which can inhibit the inflammatory response [375].

Though exosomes are heavily investigated for their therapeutic potential, they are also capable of propagating detrimental pathology in heart disease. In MI, miR-155-enriched exosomes secreted by activated macrophages were found to negatively regulates fibroblast differentiation and promote inflammation, exacerbating cardiac rupture [376]. In atherosclerosis, *Gao* et al. reported that dendritic cell-derived exosomes induce the progression of atherosclerosis by triggering inflammatory responses [377]. Taken together, although these preliminary studies on CVDs have promising results, there are still many questions that must be answered before exosomal treatment can fully achieve a useful clinical outcome (Figure 7). 

## 13. Bone Regeneration

Bone health is a rising issue and public health concern, as in the United States, approximately 850,000 people suffer from bone fractures per year [378,379], while approximately 25 million peoples are at high risk for injury due to low bone density [380,381]. In the bone remodeling and fracture healing process, osteoclasts reabsorb old or damaged bone, while new bone is synthesized by osteoblasts [381,382]. A healing cascade is initiated, leading to the recruitment of inflammatory cells, formation of new vessels, and establishment of hematoma at the fracture site [383]. The management of these bone fractures includes autologous and allogeneic transplantation; however, these procedures have a prolonged recovery time and thus increased likelihood of complications. In recent years, tissue engineering-based biomaterials and cell therapies have emerged as major players in the treatment of bone fractures. Research has identified that the biological effect of cell-based therapies is executed largely via exosomes, and as such, exosomal regulation of bone repair has become a viable therapeutic strategy. Exosomes are secreted by cell types including osteoblasts, osteoclasts, osteocytes, and bone marrow MSCs, which are known to mediate cellular communication and participate in the regulation of bone microenvironment [384,385,386,387,388,389,390]. Furthermore, numerous studies have corroborated the potential regulatory roles of exosomes in bone remodeling and fracture healing [381,382], suggesting exosomes can be utilized as individualized therapeutic strategies to promote bone tissue repair. 

Exosomes derived from various sources have been demonstrated to be beneficial in bone repair. Chief amongst them are MSC-Exos, which play a promising role in the induction of osteo-differentiational activity through microRNA regulations [391]. Exosomes obtained from adipose tissue MSCs, preconditioned with TNF-α, promote primary osteoblast differentiation by regulating the Wnt pathway [392]. Separately, exosomes derived from mineralizing osteoblast cells promote new bone growth through directly regulating osteoblast proliferation and stimulating the differentiation of osteoblast precursors into mature osteoblasts via mediating miRNA profiles. This successively activates downstream signaling pathways for bone formation and matrix mineralization [384]. Another group has reported that exosomes isolated from human-induced pluripotent stem cell-derived MSCs potently enhance osteo-inductivity of β-TCP through activation of PI3K/AKT pathway [393]. Further, this study demonstrated that in an immunocompetent rat osteochondral defect model, the MSC-Exos promote cartilage regeneration by enhancing early cellular infiltration and proliferation, inducing synovial macrophage polarization, and exhibiting anti-apoptotic activity [393]. 

Bone remodeling is a long-lasting process in which can exosomes play crucial regulatory roles. Exosomes derived from osteoblasts stimulate the differentiation of osteoclasts in vivo, and as such exosome treatment can be used to enhance the removal of damaged tissue [394,395]. It has also been revealed that in a femur fracture model of CD9^−^*/*^−^ mice, which lacks exosome production, there is a delay of callus formation resulting in remission of a bone union. Providing further support for the beneficial role of exosomes in bone repair, the administration of MSC-Exos rescued this delayed repair [396]. The same study further explained that, when compared to exosomes isolated from MSCs cultured in preconditioned media, control exosomes were deprived of bone repair-related cytokines such as monocyte chemotactic protein-1(MCP-1), MCP-3, and stromal cell-derived factor-1 [396]. In a rat osteochondral defect model, the intra-articular administration of human embryonic MSC-Exos induces an enhanced gross appearance and improved histological scores; by 12 weeks of treatment, the cartilage and subchondral bone was fully restored [397]. In addition, studies have shown that overactivated inflammatory response or inflammation attenuation results in either bone tissue damage or accumulation of necrotic tissue, respectively [398,399,400]. MSC-Exos are shown to diminish inflammation-based delay of fracture healing by effectively suppressing levels of proinflammation factors IL-1β and TNF-α, whereas increasing levels of anti-inflammatory factor TGF-β [401]. 

Vascularization is an important event in bone formation as it improves diffusion of oxygen and nutritional components that are crucial for new bone formation [402]. Various reports have demonstrated the involvement exosomes in the activities of endothelial cells (ECs) including migration, proliferation, and tube formation. Exosomes derived from endothelial progenitor cells induce the formation of vessel-like structures both in vitro and in vivo by activating eNOS and PI3K/AKT pathways in human umbilical vein endothelial cells and human mammalian ECs [403]. In addition, the contribution of MSC-Exos in angiogenesis is well established [404,405]. Researchers have shown that MSC-Exos are internalized into tissue engineering-based matrix, which results in pro-angiogenic and pro-osteogenic activities, suggesting exosomes may regulate synergistic bone regeneration and enhance angiogenesis [406]. Moreover, in an in vivo study, MSC-Exos delivered to osteoporotic rats had elevated angiogenesis and osteogenesis eight weeks post-administration [407]. However, the coupling underlying mechanisms of angiogenesis and osteogenesis is still unclear. 

A primary mechanism of action is osteopathic repair is through microRNA activity and regulation, many of which have been identified within exosomes. For example, researchers have identified that miR-199b is involved in the regulation of Runx 2, a master transcription factor for osteoblast and osteocyte differentiation [391]. Let-7 positively regulates osteogenesis and new bone formation by downregulating high-mobility group AT-hook 2 expression [408], while upregulated levels of miR-135b significantly inhibit osteogenic differentiation of MSCs [409]. In both human MSCs and human unrestricted somatic stem cells, miR-221 downregulation showed to stimulate osteogenic differentiation [410]. The crucial role of miR-21 in enhancing the rate of fracture healing has also been illustrated in a rat closed femur fracture model [411]. miR-21 rich exosomes possess anti-apoptotic properties and can contribute to osteoblastogenesis through the regulation of small mothers against decapentaplegic 7 (Smad7) and modulating PI3K/β-catenin pathways [412,413]. Exosomes can also serve as a biomarker, as altered miRNA profiles in MSC-Exos have been discovered during osteogenesis [391]. Therefore, the involvement of specialized cargo within exosomes enriched with specific factors such as miRNA, cytokines, or growth factors can play vital roles during bone repair and diagnostics [396]. This collective data and positive outcomes provided new insight for exosome-based synergetic therapy for bone fracture healing.

## 14. COVID-19

In late 2019, Chinese health authorities reported an outbreak of pneumonia of unknown origin in the city of Wuhan [414,415,416]. This led to the occurrence of a global pandemic, now known as COVID-19, resulting in thousands of deaths and infecting millions of people across the world (https://coronavirus.jhu.edu/map.html accessed 25 June 2021). COVID-19 has had an enormous destabilizing impact on society, including the healthcare sector and economies of even the most developed nations. A novel coronavirus named 2019-nCoV (later renamed as SARS-CoV-2) is known to be the causative agent and was first isolated from the airway epithelial cells of a patient [414,415,416]. SARS-CoV-2 was first sequenced in China and found to be different from previously identified viruses including MERS-CoV and SARS-CoV. It is now considered as the seventh member of the family of coronaviruses that are known to infect humans. SARS-CoV-2 is mainly transmitted via droplets within individuals of a susceptible population. It has also been shown to survive on glass and banknotes surfaces for 24–72 h [417]. However, the exact molecular mechanism of infection from SARS-CoV-2 is currently unknown. 

MSCs have been substantially investigated as useful tools for stem cell-based therapy of degenerative diseases, cardiovascular disease, and lung diseases due to properties such as high proliferation rate, high differentiation, and vast source potential. Recently, several studies have posited that COVID-19 can hijack the immune system of the body and trigger an overreaction, ultimately leading to extreme inflammation and organ damage. In COVID-19, following its infection of the lungs, SARS CoV-2 virus triggers an immune response of cytokine secretion along with other immune cells, resulting in a hyper inflammation condition called cytokine storm. Numerous studies have shown that MSCs can interact with and regulate the function of immune cells such as B cells, DCs, natural killer cells, macrophages, T cells, and neutrophils [418,419,420]. In COVID-19, the SARS-CoV-2 enters the body by binding to angiotensin-converting enzyme 2 (ACE2), an enzyme located on the membrane of many cell types, including those in the lung [421,422]. MSCs do not contain any ACE2 receptors, making them immune to SARS-CoV-2. Recent research has found that transplanting MSCs in COVID-19 patients has immunomodulatory effects that can prevent the cytokine storm and decrease the damage to tissues through the regenerative potential of MSCs [423,424]. 

**Figure 8:**
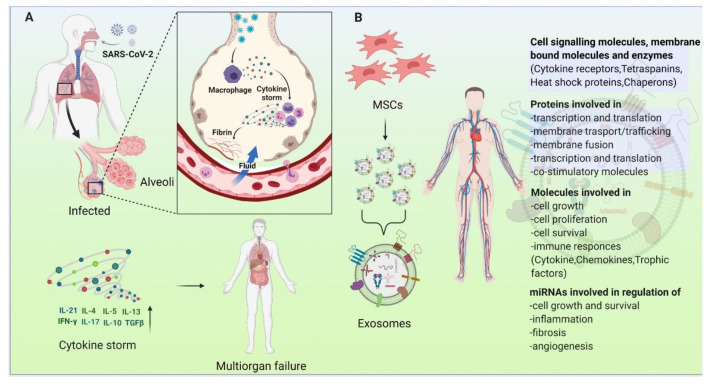
Schematic representation of role of cytokine storm in SARS-CoV-2 infection and possible therapeutic effects of MSCs and MSC-derived exosomes. (**A**) Pathogenesis of SARS-CoV-2; SARS-CoV-2 virus targets the lung epithelial cells. During this process, immune cells such as macrophages identify the virus particles and start to produce pro-inflammatory cytokines. These cytokines attract more immune cells, which in turn enhance the cytokine production. This creates a cycle of inflammation that damages the lung cells through the formation of fibrin. This damage results in weakened blood vessels, allowing fluid to seep in and fill the lung cavities, leading to respiratory failure and failure in multiple organs. MSCs and MSC-derived exosomes have the potent ability to suppress the inflammatory responses and prevent the progression towards multiple organ failure. (**B**) Putative mechanisms of MSC and MSC-derived exosome therapy in severe SARS-CoV2 cases; MSCs and MSC-derived exosomes have the potent ability to suppress the inflammatory responses and prevent the progression towards multiple organ failure. Isolated MSCs cultured in conditioned media that induces the release of exosomes. The MSCs identify the extracellular signals and start the packaging of several regulatory factors into exosomes that are released into the conditioned medium. These exosomes contain different growth factors, metabolic enzymes, molecules involved in immunomodulation, cytokine receptors, molecules important for cell division and proliferation, DNA, and regulatory miRNAs. In MSC-derived exosome therapy, exosomes are administered intravenously or via aerosol inhalation. The administered exosomes inhibit inflammatory cytokines and other immune regulatory cells which results in the suppression of the cytokine storm.

Interestingly, several studies have demonstrated that exosomes derived from MSCs suppress cytokine release and reduce the level of inflammation [418,425,426]. In addition to this, Dinh et al. showed that exosomes can help in lung regeneration in pulmonary fibrosis conditions [250]. Therefore, exosomes could possibly be used in the treatment of critically ill COVID-19 patients in combination with other therapies (Figure 8). To date, more than 90 MSCs based studies for respiratory diseases have been registered for clinical trials (https://clinicaltrials.gov, accessed 25 June 2021). In addition, Ruijin hospital in China registered a pilot clinical study to explore the inhalation of exosomes derived from allogenic adipose MSCs in patients critically ill with COVID-19 (ClinicalTrials.gov Identifier: NCT04276987). Similarly, several other, similar clinical trial studies have been registered worldwide (Table 9). Therefore, there is growing evidence that the use of MSC-Exos in COVID-19 therapy may limit the respiratory complications in patients.

## 15. Conclusions and Perceptive

In recent years, it has been established that exosomes have modulatory potential and play a critical role in diverse biological processes. Exosomes show tremendous therapeutic potential for disorders including chronic wound healing, neurological damages, and cardiovascular dysfunction. Exosomes have also gained widespread attention in the field of biomarker research and are now even being seen as an alternative strategy to stem cell-based regenerative therapies. Exosomes can be genetically engineered to deliver distinct therapeutic moieties to a desired target. These cargos include recombinant proteins, antagomirs, short interfering RNAs, antisense oligonucleotides, and immune modulators [427]. 

Although exosomes have attained significant achievements in several therapies, challenges remain. While numerous proteins, RNAs, lipids, and metabolic enzymes (Table 2, Table 3 and Table 4) have been identified in exosomes, little is known about their functions and sorting mechanisms. Exosomal cargo is also highly dependent on surrounding milieu and metabolic status of host cells. It remains ambiguous whether natural, physiological levels of small vesicles exert any pathological or regulatory roles in vivo. Despite several exosomal studies in chronic wound healing and skin regeneration, the exact molecular mechanism and the role of exosomes in these processes require further investigations. Additionally, for better exploitation of exosomes, an extensive study is needed in the areas of biogenesis, cellular uptake, and trafficking of exosomes. Moreover, other critical challenges involving the use of exosomes in regenerative medicine require further investigations, such as isolation, purification, optimization, standardization, quality control, and further exploration of molecular mechanisms of exosome communication with target cells. Overall, the potential of exosomes derived from various sources in regenerative medicine and tissue engineering is highly promising.

## Figures and Tables

**Table 1:** Composition and function of complexes and key enzymes related to exosome biogenesis, sorting, transport, and secretion.

Complexes	Subunits	Localization	Function	References
ESCRT	ESCRT-O (HRS, STAM)	MVBs	Recognizes and binds ubiquitinated proteins and sorts them into spatially restricted areas on the endosomal membrane; HRS recognizes the mono-ubiquitinated proteins and recruits TSG101	[78,79,88]
ESCRT	ESCRT-1 (TSG101, VPS28, VPS37, MVB12); ESCRT-2 (VPS36, VPS22, VPS25)	MVBs	Regulates the initial deformation of membrane into buds with sequestered cargo and may be involved in cargo transfer	[78,79,88]
ESCRT	ESCRT-3 (VPS2, VPS20)	ILV, MVBs	Drives membrane invagination and subsequent vesicle scission	[78,79,88]
AAA ATPases	VPS4	ILVs	Interact with ESCRT-3 to cause constriction and scission of ILV	[88]
ESCRT-associated proteins	ALIX	ILVs, MVBs	Controls exosomal cargo incorporation and regulates sorting of PD-L1 on to ILVs; ALIX and syntenin-ALIX and syntenin-ALIX complex stimulate intraluminal budding	[82,86]
Rabs	Rab5	PM, Early endosome	Mediates endocytosis and generation and maintenance of early endosomes	[80,87]
Rabs	Rab7	MVBs	Mediates maturation and trafficking of MVBs to lysosomes	[87]
Rabs	Rab27a	MVBs	Involved in the fusion of MVBs to the PM	[83,85]
Rabs	Rab27b	MVBs	Promotes formation and stability of MVB docking and facilitates exosome shedding	[83,84]
Rabs	Rab35	MVBs	Controls MVB transport and influences the docking process	[89]
SNAREs	t-SNARE; v-SNARE	Widespread distribution in endosomal system	Drive membrane fusion and mediate fusion of MVBs with the PM	[89,90]
Enzyme	Heparanase	PM, endosome membrane	Exogenous heparanase impacts intraluminal budding and, therefore, exosome biogenesis	[86]
Enzyme	snMase2/SMPD3	PM, endosomes	Regulates biosynthesis of ceramide and promotes budding of intravesicular vesicles	[81,85]

Abbreviation: ESCRT, endosomal sorting complex required for transport; STAM, signaling transducing adaptor molecule; MVB, multivesicular body; VPS, vacuolar protein sorting associated protein; ILV, intraluminal vesicles; ALIX, apoptosis-linked gene 2-interacting protein X; PM, plasma membrane.

**Table 2:** Distinct protein families identified in exosomes from different cell types.

Protein Class	Name	Cell Type	Reference
**Antigen Presentation**	MHC class I	B cells Dendritic cells Enterocytes Tumors T cells	[103,104,105,106,107]
	MHC class II	B cells Dendritic cells Enterocytes Mastocytes T cells	[74,103,105,107,108]
	CD86	Dendritic cells	[109]
**Integrins**	α4β1	Reticulocytes	[110]
	αMβ2	Dendritic cells	[111]
	β2	T cells	[103]
	αLβ2	Mastocytes	[112]
	α3		
**Immunoglobulin family members**	ICAM1/CD54	B cells Dendritic cells Mastocytes	[112,113,114]
	P-selection	Platelets	[112]
	A33 antigen	Enterocytes	[105]
**Cell surface peptidases**	Dipeptidylpeptidase IV/CD26	Enterocytes	[105]
	Aminopeptidase n/CD13	Mastocytes	[112]
**Tetraspanins**	CD63	B cells Dendritic cells Enterocytes Mastocytes T cells Platelets	[103,105,107,114,115,116]
	CD37, CD53, CD81, CD82	B cells	[114]
	CD9	Dendritic cells	[111]
**Heat shock proteins**	HSC70	Tumors Reticulocytes Dendritic cells	[16,106,111]
	HSP70	Tumors Peripheral blood mononuclear cells	[117,118,119,120]
	HSP84/90	Enterocytes Dendritic cells	[105,111]
**Cytoskeletal proteins**	Actin	Mastocytes Dendritic cells Enterocytes	
	Actin binding protein (cofilin)	Dendritic cells	
	Tubulin	Dendritic cells Enterocytes	
**Membrane transport and fusion**	Annexins I, II, IV, V, VI	Dendritic cells	[121]
	Annexin VI	Mastocytes	[112]
	RAB7/RAP1B/RABGDI	Dendritic cells	[121]
**Signal transduction**	Gi2α/14-3-3	Dendritic cells	[121]
	CBL/LCK	T cells	[103]
**Metabolic enzymes**	Enolase 1	Enterocytes	[105]
	Thioredoxin peroxidase	Dendritic cells	[121]

**Table 3:** Important protein families identified within or externally located on exosomes [122,123,124,125].

Protein Class	Name
**Metabolic enzymes**	Phosphoglycerate kinase 1
	NADP-dependent malic enzyme
	Citrate synthase
	Malate dehydrogenase
	Fatty acid binding protein-3
	Fatty acid binding protein-4
**Secretory/membrane proteins**	MFG-E8
	Adiponectin
	Fasting-induced adipose factor
	CD9
	CD63
	Integral membrane protein TAPA-1
	Clathrin heavy chain
	MMP-2
	MMP-9
	Coagulation factor II
	Coagulation factor V
	Fibulin 2
	Annexin II
**Heat shock proteins**	Hsp1
	HSP60
	HSP70
	HSP75
	HSP84
	TCP-1 chaperone family β-subunit
	TCP-1 chaperone family γ-subunit
	TCP-1 chaperone family ε-subunit
**GPI-anchored proteins**	Gce1
	CD73
**Ubiquitin/proteasome-related proteins**	26S proteasome subunit α1
	26S proteasome subunit β3
	26S proteasome subunit β5
	26S proteasome subunit Rpn13
	Ubiquitin-activating enzyme E1
**Nuclear proteins**	Histone H1.1
	Histone H1.5
	Histone H1.3
	Histone H2A
	Histone H2B
	Histone H4
	40S ribosomal protein SA
	40S ribosomal protein S8
	60S ribosomal protein L7
	60S ribosomal protein L18a
**Cytoskeleton**	Actr1b protein
	β-Actin
	Tubulin α2
	Tubulin β2
	Tubulin β5
**Membrane transport/fusion**	Annexin II
	Annexin V
**Miscellaneous**	Galectin 1
	Galectin 3
	Translation elongation factor-1
	Translation elongation factor-2
	Translation initiation factor 4A
	Major vault protein
	14-3-3β
	Dynein heavy chain
	Albumin

**Table 4:** Lipid families identified in exosomes released from various cell types.

Lipids	Cell Types	References
SM, PC, PE, PS, PI, CHOL, Plip	Erythrocytes	[126]
SM, PC, PE, PS, PI, LPC, CHOL, Plip, HexCer, LacCer	Mast cells	[70,126,127]
SM, PC, PE, PS, PI, LPC	Dendritic cells	[126]
SM, GM3, PCP, SPI, PE, EthLip, CholPlip, DAG	B lymphocytes	[70,126]
SM, PC, PS, PE, CholPlip	T lymphocytes	[70,126]
Acylcarnitine, Cholesterol, Sphingolipids, glyserolipids, glycerophospholipids	Non-tumorigenic (RWPE1), tumorigenic (NB26) and metastatic (PC-3) prostate cell lines	[128]
CHOL, SM, PC, PS, PE, PE, ethers, DAG, PC ethers, PG, PA, PI, Cer, HexCer, LacCer	PC-3 Cells	[70,92]
CHOL, SM, PC, PS, PE, PE, ethers, DAG, PC, ethers, PG, PA, PI, Cer, HexCer, LacCer	PC-3 cells + HG	[70]
CHOL, SM, PC, PS, PE, PI, Cer, HexCer, LacCer	Oli-neu Cells	[70]
CHOL, SM, PC, PS, PE, PI, CerHex, Cer, LacCer	HepG2/C3a	[70,129]
CHOL, SM, PC, PS, PE, PI, Cer, HexCer, LacCer, Gb3	Prostasome	[70,130]
CHOL, SM, PC, PS, PE, PE, ethers, PC, ethers PI, Cer, HexCer, LacCer, Gb3	Urine	[70,131]
CHOL, SM, PC, PS, PE, PE, ethers, PC, ethers	Nematodes	[70,132]
CHOL, SM, PC, PS, PE, PI, Cer, HexCer, LacCer	Reticulocytes	[71,73]
CHOL, SM, PC, PS, PE, PE, ethers, PC, ethersPI, Cer	Platelets	[71,133]
CHOL, SM, PC, PS, PE, PE, ethers, DAG, PI, CerHexCer	Adipocytes	[71,134]

Abbreviation: EthLip, ether lipids; CHOL, cholesterol; PLIP, phospholipids; Prot, proteins; SM, sphingomyelins; PC, phosphatidylcholines; PE, phosphatidylethanolamines; PS, phosphatidylserines; PI, phosphatidylinositols; LPC, lysophosphatidylcholines; PE, Phosphatidylethanolamine; PG, Phosphatidylglycerol; PA, Phosphatidic acid; SMG, Sphingomyelin; GSLe, lycosphingolipids; CE, Cholesterol ester; LPCb, Lysophosphatidylcholine; DAG, diacylglycerol; LacCer, lactosylceramide.

**Table 5:** Characteristics of exosome isolation methods [3,5,168,169].

	Isolation Technique	Equipment	Isolation Principle	Advantages	Disadvantages
**Traditional methods**	Ultra-centrifugation	Ultra-centrifuge	Physical method	High sample capacity; Protein and RNA components are not affected; Facilitated later research	Time-consuming; Instrument-dependent; Low purity
	Density gradient	Ultra-centrifuge	Physical method	High separation efficiency; High purity; Exosomes will not be crushed or deformed	Extended run time; Equipment dependence; Low yield; Complex process
	Immuno-magnetic beads	Magnetic bead, antibody	Chemical method	Time efficient; Maintain integrity; Convenient operation; Not affected by exosome size; No need for expensive instruments	High reagent cost; Low capacity; Low yields
	Precipitation	Ultra-centrifuge	Physical/ Chemical method	High yield; Easy; Concentrates diluted samples	Post-clean up is needed for downstream applications
**Emerging methods**	ExoQuick	ExoQuick kit	Physical/ Chemical method	Simple steps, Quick operation; Size uniformity; Suitable for small samples, such as serum	Impurity; Affected by exosome diameter; Expensive reagents; Low production
	Size Exclusion Chromatography	Gel filtration column	Physical/ Chemical method	High purity; Uniform in size	Low extraction volume; Extensive laboratory equipment requirements
	Stirred ultrafiltration	Ultra-filtration membrane, Nitrogen gas	Physical method	Does not rely on equipment; Less time consuming than other methods; Reduces the destruction of exosomes during the process	Moderate purity of isolated exosomes; Loss of exosomes during the process
	Filtration Device	Microfluidic devices (e.g., nano traps)	Physical/ Chemical method	Fast, Low cost; Easy automation and integration; High portability	Lack of standardization and large-scale tests on clinical samples, Lack of method validation; Low sample capacity
	nPES	GNPs, Antibodies	Chemical method	Fast; Efficient; High purity; Quantitative analysis	High reagent cost; Complex statistical tools; Low capacity
	Membrane modification	Magnetic field, Magnetic nanoparticles	Physical/ Chemical method	Needs no antibodies; Save time; preserve the original structure of theexosomes; Drug carriers	Complicated operation; Impurity
	ExoTIC	ExoTIC, Syringe, Pump	Physical/ Chemicalmethod	Simple operation; Exosomes in a specific range; High purity	Special equipment requirements; Lack of tests on clinical samples
	Flow field-flow fractionation	Flow field-flow fractionation instrument	Physical method	Label free isolation; Large scale production	Special instrument requirement; Costly

**Table 6:** Summary of major growth factors families and their applications in injury healing.

Growth Factor	Source	Molecular Function	References
**VEGF**	Keratinocytes, Fibroblasts, Macrophages, Endothelial cells Smooth muscle cells	Inflammation, Angiogenesis	[200,213,214,215,216,217,218]
**CX3CL1**	Macrophages, Endothelial cells	Inflammation, Angiogenesis, Collagen deposition	[219,220]
**TGF-β**	Fibroblasts, Keratinocytes, Macrophages, Platelets	Inflammation, Angiogenesis, Granulation tissue formation, Collagen synthesis, Tissue remodeling, Leukocyte chemotactic function	[200,217,221,222,223,224,225,226,227,228]
**IL-6**	Fibroblasts, Endothelial cells, Macrophages, Keratinocytes	Inflammation, Angiogenesis, Re-epithelialization, Collagen deposition, Tissueremodeling	[217,229,230,231]
**IL-1**	Macrophages, Leukocytes, Keratinocytes, Fibroblasts	Inflammation, Angiogenesis, Re-epithelialization, Tissue remodeling	[217,232,233,234,235]
**PDGF**	Platelets	Inflammation, Re-epithelialization, Collagen deposition, Tissue remodeling	[212,236]
**IL-27**	Macrophages	Suppression of inflammation, Collagen synthesis	[237,238]
**HGF**	Fibroblasts	Suppression of inflammation, Granulationtis sue formation, Angiogenesis, Re-epithelialization	[215,217,239]
**Activin**	Keratinocytes, Fibroblasts	Granulation tissue formation, Keratinocyte Differentiation, Re-epithelialization,	[240,241,242,243]
**FGF-2**	Keratinocytes, Fibroblasts, Endothelial cells	Angiogenesis, Granulation tissue formation	[217,244,245,246,247,248]
**Angiopoietin-1/-2**	Fibroblasts	Angiogenesis	[216,249]
**EGF, HB-EGF, TGF-α**	Keratinocytes, Macrophages	Re-epithelialization	[217,250,251,252,253,254]
**FGF-7, FGF-10**	Fibroblasts, Keratinocytes	Re-epithelialization, Detoxification of ROS	[200,217,255,256,257]
**CXCL10, CXCL11**	Keratinocytes, Endothelial cells	Re-epithelialization, Tissue remodeling	[258,259,260]
**IL-4**	Leukocytes	Collagen synthesis	[217,226]
**GM-CSF**	Macrophages, T cells, Mast cells, Natural killer cells, Fibroblast, Endothelial cells	Recruit Langerhans cells, Stimulate proliferation and differentiation	[239,261]
**TNF-α**	Neutrophils Macrophages	Inflammation Re-epithelialization	[262]

Abbreviation: CXCL10/11, cysteine-X amino acid-cysteine; EGF, epidermal growth factor; FGF, fibroblast growth factor; HB-EGF, heparin binding EGF; HGF, hepatocyte growth factor; IL, interleukin; PDGF, platelet-derived growth factor; TGF, transforming growth factor; VCAM-1, vascular cell adhesion molecule-1; VEGF, vascular endothelial growth factor.

**Table 7:** List of miRNAs involved in the various stages of wound healing. Highly expressed microRNA are bolded [158,160,279,280,281,282,283,284].

Inflammatory Phase	Proliferation Phase			Remodeling Phase	Migration	Invasion
	Re-Epithelialization	Angio-Genesis	Granulation Tissue Formation			
miR-17-5p	**miR-21**	miR-1	miR-15a	**miR-29a**	**miR-196a**	miR-141
miR-18a	**miR-31**	**miR-21**	miR-15b	**miR-29b**		miR-200b
miR-34a	miR-203	**miR-23a**	miR-16	miR-29c		miR-200c
miR-106b	miR-204	miR-29b	miR-17	miR-192		
**miR-181a**	miR-205	miR-126	miR-17-92			
miR-181b	miR-210	miR-133a	miR-20a			
miR-193b		miR-133b	miR-20b			
miR-210		miR-146a	**miR-21**			
**miR-221**		miR-210	miR-29			
**miR-222**		miR-218	**miR-92a**			
		miR-377	miR-98			
		miR-939	miR-101			
		miR-4530	**miR-126**			
			**miR-130a**			
			miR-141			
			miR-184			
			miR-185			
			miR-200b			
			miR-203			
			miR-205			
			miR-206			
			miR-210			
			miR-221			
			miR-222			
			miR-296			
			miR-320a			
			miR-320b			
			miR-378a			

**Table 8:** MSC-derived exosomes in wound healing therapies.

Study	MSC Source	Particle Isolation	Particle Size	MSC Exosome Characterization	Model Species/Cells	Intervention(s), Route, and Dose	Important Finding from the Studies
(Choi et al., 2019) [299]	Human adipose-derived (AD) MSCs	Centrifugation, TFF	133 ± 14 nm	EM, WB, NTA	UV damaged Human dermal fibroblasts	1 × 10^8^ particles in 1 mL PBS	Increased collagen and elastin synthesis and decreased MMP activity
(Hu et al., 2016) [267]	Human ADMSCs	Ultrafiltration and ExoQuick-TC kit	30–100 nm	EM, WB, NTA	Adult male Balb/c mice	Injection of 200 μg of total exosome protein in 200 μL PBS	Increased wound healing by regulating the migration, proliferation, and collagen synthesis of fibroblast
(Ferreira et al., 2017) [300]	Human ADMSCs	UC	135 nm	NTA	Human epidermal keratinocytes and dermal fibroblasts	Exosome-gel transplantation/1.9 × 10^8^ particles in hydroxyethyl cellulose aqueous gel	Enhanced wound healing through increased migration and proliferation of keratinocytes and fibroblasts
(Cooper et al., 2018) [304]	Human ADMSCs	ExoQuick-TC kit	90–100 nm	NTA	Six-month-old male Fischer 344 rats	Injection of conditioned media from ZenBio™ ADSC (20 μL) or control (unconditioned) media (20 μL)	Increased wound healing through increased migration and proliferation of keratinocytes and fibroblasts
(Shi et al., 2020) [297]	Human ADMSCs	UC	50–120 nm	EM, WB	Male C57BL mice	Subcutaneous injection of exosomes/200 μg in 100 μL of PBS	Enhanced wound healing through inducing miR-128-3p/SIRT1-mediated autophagy
(Shabbir et al., 2015) [48]	Human bone-marrow (BM) MSCs	UC	30–100 nm	EM, WB, NTA	Human dermal fibroblasts	0.1, 1, and 10 μg total exosome protein in 1 mL; PBS/details not provided	Increased migration and proliferation of fibroblasts and tube formation by endothelial cells
(Ding et al., 2019) [268]	Human BMMSCs	UC	50–150 nm	EM, WB, NTA	Adult male Sprague–Dawley (SD) rats	Subcutaneous injection of exosomes/100 μg in 100 μL of PBS	Enhanced wound healing through elevated proaniogenic activity
(Zhang et al., 2016) [298]	Human umbilical cord (UC) MSCs	Differential centrifugation and sucrose gradient UC	30–100 nm	EM, NTA	Adult female SD rats	Injection of 200 μg of total exosome protein in 200 μL PBS	Increased wound healing through increased proliferation, angiogenesis of fibroblasts
(Fang et al., 2016) [305]	Human UCMSCs	Differential centrifugation and sucrose gradient UC	30–100 nm	WB, NTA	Adult male ICR mice (Swiss-Hauschka mice) and nude mice (BALB/c-ν)	Exosome-scaffold transplantation (HydroMatrix)/100 μg of total exosome protein in 100 μL PBS	Enhanced wound healing through supression of myofibroblast differentiation
(Ti et al., 2015) [306]	Human UCMSCs	UC	40–90 nm	EM, WB	Diabetic rats (Details not provided)	Injection at wound site/MSC-exosomes (60 μg) and LPS-preconditioned MSC-exosomes (60 μg)	Increased diabetic cutaneous wound healing through regulation of macrophage polarization and resolution of chronic inflammation
(Zhang et al., 2015) [50]	Human iPSC-MSCs	UC	30–100 nm	EM, FC, WB	Adult male SD rats	Injection/160 μg total exosome protein in 160 μL; PBS at wound sites and 40 μg total exosome protein in 40 μL PBS at wound beds; 14-day study	Enhanced angiogenesis and collagen synthesis
(Bian et al., 2020) [307]	Human decidua	UC	64–125 nm	EM, WB, NTA	Female diabetic mice (BKS-Dock Leprem2Cd479, db/db)	dMSC-s Exosomes (100 μL, 5.22 × 1011 particles/mL) injected around wounds weekly for 4 weeks	Enhanced human dermal fibroblasts migration, proliferation, and differentiation and improved senescent state
(Li et al., 2016) [302]	Human synovium	UC	85 ± 36 nm	EM, DLS, WB	Adult male SD rats	Hydroxyapatite/chitosan composite hydrogels loaded with exosomes	Increased wound healing through re-epithelialization, accelerating angiogenesis, and expediting collagen maturity

Abbreviations: BM, bone-marrow; MSC, mesenchymal stem cells; iPSC, induced-pluripotent stem cells; AD, adipose-derived; UC, umbilical cord; UC, ultracentrifugation; EM, electron microscopy; WB, western blot; NTA, Nanoparticle tracking analysis; FC, flow cytometry; DLS, dynamic light scattering; TFF, tangential flow filtration; SD, Sprague-Dawley; PBS, Phosphate Buffered Saline; MMP, matrix metalloproteinase.

**Table 9:** List of registered exosome cell based clinical trials for the treatment of COVID-19.

Registration Number	Study Topic	Cell Source	Type of Study
**NCT04276987**	A Pilot Clinical Study on Inhalation of Mesenchymal Stem Cells Exosomes Treating Severe Novel Coronavirus Pneumonia	Adipose MSC-derived exosome	Biological: MSCs-derived exosomes
**NCT04493242**	Extracellular Vesicle Infusion Therapy for Severe COVID-19 (EXIT COVID-19)	Bone marrow derived extracellular vesicles.	Intervention study
**NCT04389385**	COVID-19 Specific T Cell Derived Exosomes (CSTC-Exo)	COVID-19 Specific T Cell derived exosomes (CSTC-Exo)	Intervention study
**NCT04384445**	Zofin (Organicell Flow) for Patients With COVID-19	Human amniotic fluid derived exosomes	Intervention study
**NCT04902183**	Safety and Efficacy of Exosomes Overexpressing CD24 in Two Doses for Patients With Moderate or Severe COVID-19	-	Intervention study
**NCT04747574**	Evaluation of the Safety of CD24-Exosomes in Patients With COVID-19 Infection	-	Intervention study
**NCT04491240**	Evaluation of Safety and Efficiency of Method of Exosome Inhalation in SARS-CoV-2 Associated Pneumonia. (COVID-19EXO)	-	Intervention study
**NCT04798716**	The Use of Exosomes for the Treatment of Acute Respiratory Distress Syndrome or Novel Coronavirus Pneumonia Caused by COVID-19 (ARDOXSO)	MSC-exosomes	Intervention study
**NCT04657406**	Expanded Access to ZofinTM (OrganicellTM Flow) for Patients With COVID-19	MSC-exosomes	Intervention study
**ChiCTR2000030484**	Clinical application of umbilical cord mesenchymal stem cells combined with intravenous exosome infusion to repair lung injury of new coronavirus pneumonia (COVID-19)	Adipose MSC-derived exosome	Intervention study
**ChiCTR2000030261**		Bone marrow derived extracellular vesicles.	Intervention study

## Data Availability

No new data were created in this study. Data sharing is not applicable to this article.
